# TRPV4 Activation during Guinea Pig Airway Smooth Muscle Contraction Promotes Ca^2+^ and Na^+^ Influx

**DOI:** 10.3390/ph17030293

**Published:** 2024-02-24

**Authors:** Luis M. Montaño, Abril Carbajal-García, María F. Casas-Hernández, David Arredondo-Zamarripa, Jorge Reyes-García

**Affiliations:** Departamento de Farmacología, Facultad de Medicina, Universidad Nacional Autónoma de México, Mexico City 04510, Mexico; lmmr@unam.mx (L.M.M.); carbajalabril@gmail.com (A.C.-G.); fercasashdz@hotmail.com (M.F.C.-H.); david.arredondo.zamarripa@gmail.com (D.A.-Z.)

**Keywords:** TRPV4, airway smooth muscle, intracellular Ca^2+^ concentration, smooth muscle contraction, asthma

## Abstract

Airway smooth muscle (ASM) contraction is determined by the increase in intracellular Ca^2+^ concentration ([Ca^2+^]_i_) caused by its release from the sarcoplasmic reticulum (SR) or by extracellular Ca^2+^ influx. Major channels involved in Ca^2+^ influx in ASM cells are L-type voltage-dependent Ca^2+^ channels (L-VDCCs) and nonselective cation channels (NSCCs). Transient receptor potential vanilloid 4 (TRPV4) is an NSCC recently studied in ASM. Mechanical stimuli, such as contraction, can activate TRPV4. We investigated the possible activation of TRPV4 by histamine (His)- or carbachol (CCh)-induced contraction in guinea pig ASM. In single myocytes, the TRPV4 agonist (GSK101) evoked an increase in [Ca^2+^]_i_, characterized by a slow onset and a plateau phase. The TRPV4 antagonist (GSK219) decreased channel activity by 94%, whereas the Ca^2+^-free medium abolished the Ca^2+^ response induced by GSK101. Moreover, GSK101 caused Na^+^ influx in tracheal myocytes. GSK219 reduced the Ca^2+^ peak and the Ca^2+^ plateau triggered by His or CCh. TRPV4 blockade shifted the concentration–response curve relating to His and CCh to the right in tracheal rings and reduced the maximal contraction. Finally, the activation of TRPV4 in single myocytes increased the Ca^2+^ refilling of the SR. We conclude that contraction of ASM cells after stimulation with His or CCh promotes TRPV4 activation, the subsequent influx of Ca^2+^ and Na^+^, and the opening of L-VDCCs. The entry of Ca^2+^ into ASM cells via TRPV4 and L-VDCCs contributes to optimal smooth muscle contraction.

## 1. Introduction

The airway smooth muscle (ASM) spans from the trachea to the terminal bronchioles. This contractile tissue plays a crucial role in the lungs, anatomically and functionally [1,2,3]. In response to physiological and pathological stimuli, ASM contraction influences the diameter and resistance of the airways by regulating airflow, thereby enhancing or impairing ventilation [2,4,5,6]. Moreover, it also assists with defensive reflexes, such as coughing [5,7]. Numerous lung disorders, such as bronchiectasis, chronic obstructive pulmonary disease (COPD), and asthma, have been associated with the dysfunction of the ASM [2,8,9,10,11,12]. Alterations in the number and size of ASM cells and their contractile activity (i.e., excessive constriction and reduced relaxation) can lead to airway remodeling, hyperreactivity, and inflammation [9,10,11,13]. Several mechanisms are responsible for these changes, including signaling pathways mediated by G protein-coupled receptor (GPCR) and nonselective cationic channels (NSCCs) such as store-operated Ca^2+^ (SOC) channels, transient receptor potential (TRP) channels, and stretch-activated channels [5,13,14,15,16,17,18,19].

ASM contraction is driven by the activation of the myosin light chain kinase (MLCK) via a process dependent on the increase in intracellular Ca^2+^ concentration ([Ca^2+^]_i_), whereas the restoration of [Ca^2+^]_i_ basal levels and dephosphorylation of the myosin light chain via myosin phosphatase (MP) relaxes ASM [20,21,22,23,24,25,26]. The activation of specific GPCRs and downstream signaling cascades mediates the contraction of the ASM in response to the release of contractile neurotransmitters or other contractile agonists, including acetylcholine (Ach), histamine (His), leukotrienes (LTs), serotonin, ATP, and others [17,19,27,28,29,30]. Upon exposure to an agonist, the subsequently stimulated phospholipase C (PLC) hydrolyzes phosphatidylinositol 4,5-bisphosphate (PtdIns4,5P2) to produce inositol 1,4,5-trisphosphate (IP_3_) and diacylglycerol (DAG) [31,32]. In the sarcoplasmic reticulum (SR) of the ASM cells, IP_3_ receptors are activated, resulting in a transient Ca^2+^ peak followed by a Ca^2+^ plateau at lower concentrations [4,30,32]. The primary mechanism for the onset of the contractile response in ASM is the initial transient Ca^2+^ peak. In contrast, sustained contraction depends on phosphorylation processes at this low [Ca^2+^]_i_ plateau, which is maintained by influx through membrane Ca^2+^ channels, including L-type voltage-dependent Ca^2+^ channels (L-VDCCs) and NSCCs [4,30,33,34,35,36,37]. Based on the cellular mechanisms that activate them, NSCCs can be divided into SOC channels, which allow cations to pass in response to a decrease in SR Ca^2+^ content, and receptor-operated (ROC) channels, which open in response to secondary messengers (e.g., DAG) after the agonist (e.g., Ach or His) has occupied its receptor and triggered a signaling pathway [4,35,38]. NSCCs allow Ca^2+^ and Na^+^ influx into the cytoplasm of ASM cells. Na^+^ influx causes membrane depolarization, activating L-VDCCs, and increasing [Ca^2+^]_i_ [4,38,39]. Ca^2+^ influx via NSCCs and L-VDCCs contributes to the sustained contraction of ASM and the refilling of Ca^2+^ of the SR to maintain the internal Ca^2+^ stores in an optimal state to perpetuate contractions [34,35,40,41,42,43].

Transient receptor potential channels are integral proteins to most NSCCs [44]. Due to their polymodal activation, TRP channels are implicated in several physiological processes, including those that control osmotic pressure, fluid secretion, inflammation, cell adhesion, contraction, proliferation, differentiation, migration, and apoptosis [45,46,47,48]. In respiratory diseases, such as asthma, there is increasing recognition of the involvement of mechanosensitive signaling pathways in the abnormal regulation of contraction of ASM cells [48,49,50]. Of particular interest is the TRP-vanilloid 4 (TRPV4) channel. TRPV4 is a multimodal Ca^2+^/Na^+^ permeable ion channel that can be activated by osmotic and mechanical stimuli (such as cell contraction), warm temperatures (>24 °C), phorbol derivatives, arachidonic acid, and its metabolites [47,49,50,51].

Jia and colleagues obtained one of the first insights into the role of TRPV4 in ASM contraction [51]. They showed that hypotonic solutions and the TRPV4 ligand, 4-phorbol-12,13-didecanoate (4-PDD), increased intracellular Ca^2+^ levels in human ASM cells. The authors also found that hypotonic stimulation of isolated human and guinea pig airways triggered smooth muscle contraction via a mechanism that relies on membrane Ca^2+^ channels [51]. In addition, it has been suggested that TRPV4 interacts with the IP_3_ receptor (IP_3_R) to promote the activity of the Na^+^/Ca^2+^ exchanger (NCX) to regulate the intracellular Ca^2+^ concentration [52]. Moreover, pharmacologic activation of TRPV4, with the synthetic agonist GSK1016790 (here and after, GSK101), triggered contraction of human and guinea pig airways, and this contraction response was blocked with the synthetic antagonists of TRPV4, GSK2334775, and GSK2193874A (here and after, GSK219) [53]. Furthermore, an increase in [Ca^2+^]_i_ due to the activation of TRPV4 caused the release of ATP from human ASM cells, which triggered the stimulation of P2X4 receptors on mast cells and the further release of LTs and ASM contraction [47].

It is worth noting that some asthma patients are hypersensitive to hypotonic stimuli, and a single nucleotide polymorphism of the TRPV4 gene is associated with a significant decline in lung function after the administration of hypotonic saline, contributing to the development of osmotic hyperreactivity [54]. Furthermore, TRPV4 polymorphisms have also been linked to the development of COPD [55]. In addition, TRPV4 has been implicated in the inflammatory response of the airways and the proliferation of ASM cells, which may contribute to the pathogenesis of asthma and COPD [17,46,48,56,57,58].

Because ASM contraction is partially regulated by Ca^2+^ influx via NSCCs, it is believed that mechanosensitive channels such as TRPV4 may play a role in dysregulated ASM contraction and asthma. Therefore, in this study, we aimed to investigate the impact of TRPV4 activation during bronchoconstrictor agonist-induced guinea pig tracheal smooth muscle contraction. We also aim to determine the possible Na^+^ influx through the TRPV4 channel and its role in opening L-VDCCs. Our findings can potentially enhance our understanding of the role of TRPV4 in ASM physiology.

## 2. Results

### 2.1. Intracellular Ca^2+^ Increase Mediated by TRPV4 Activation with GSK101 Depends Exclusively on External Ca^2+^ Influx

**Figure 1:**
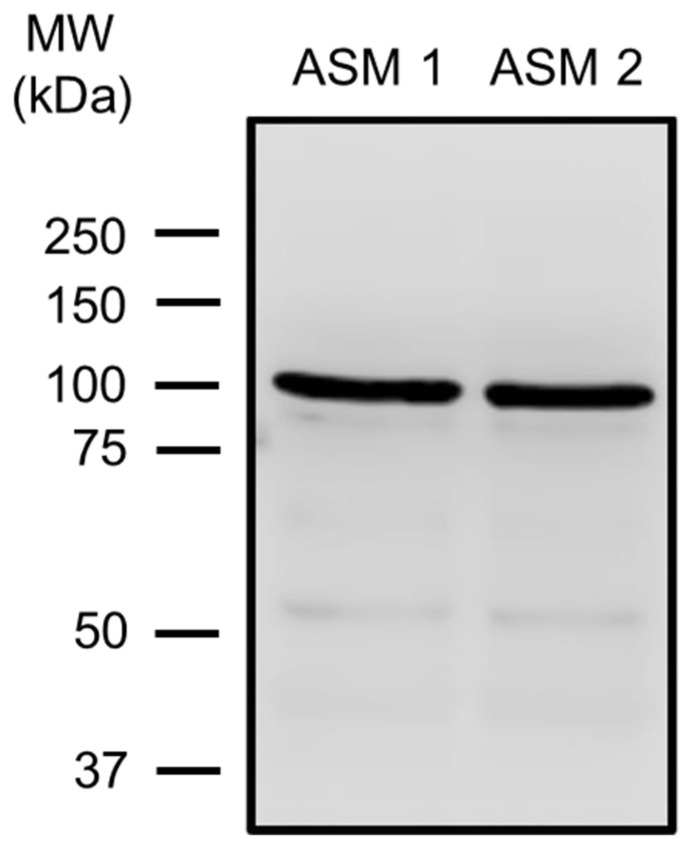
TRPV4 expression in guinea pig airway smooth muscle (ASM). Tracheal smooth muscle from two animals (ASM 1, ASM 2) was collected and dissected free from epithelium and connective tissue. Total protein was extracted, and electrophoresis (SDS-PAGE) was performed with 25 µg of total protein. A TRPV4-specific antibody (#ACC-034) was used, followed by an HRP-conjugated secondary antibody. The molecular weight (MW) is given according to the information in the commercial data sheets of the antibody. ASM 1 and ASM 2 show the blots illustrating one band around 100 kDa corresponding to TRPV4. Each blot represents a tissue from two different animals.

**Figure 2:**
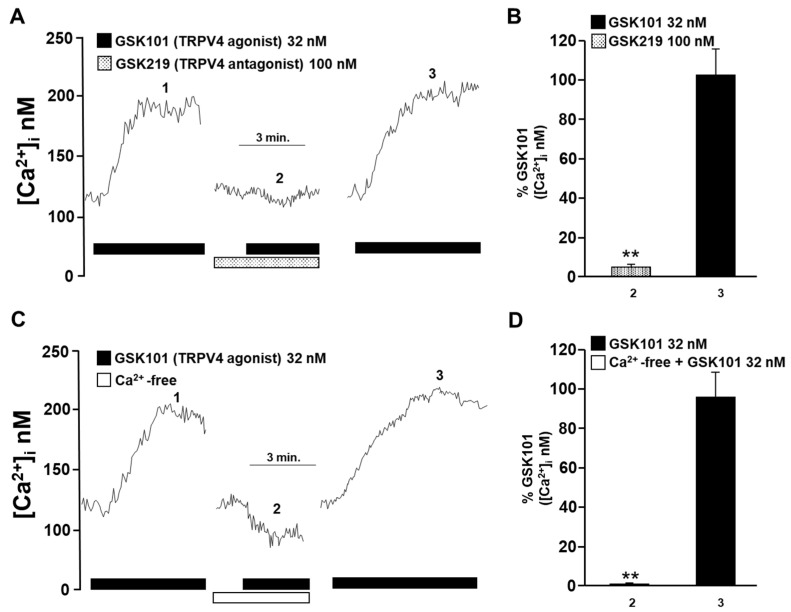
The increase in intracellular Ca^2+^ concentration ([Ca^2+^]_i_) in guinea pig tracheal myocytes induced by TRPV4 stimulation depends on the influx of Ca^2+^ from the extracellular medium. (**A**) Tracheal smooth muscle cells were constantly perfused with Krebs solution at 37 °C. The addition of 32 nM GSK101 (a selective TRPV4 agonist) increased the [Ca^2+^]_i_ ~70 nanomoles, with a slow onset and plateau phase (1). Preincubation of cells with the selective TRPV4 antagonist (GSK219, 100 nM) reduced the increase in the [Ca^2+^]_i_ caused by a second stimulation with GSK101 (32 nM) by ~94% (2). After the TRPV4 antagonist was washed out, a third stimulation with 32 nM GSK101 caused a Ca^2+^ response that was similar to the first stimulation (3). (**B**) The graph summarizes the percentage of Ca^2+^ response caused by stimulation of TRPV4 in the presence and after using the antagonist GSK219, 100 nM. ** *p* < 0.01 compared with the control group (the first stimulus with 32 nM GSK101). (**C**) Perfusion of TRPV4 agonist in a Ca^2+^-free medium did not increase [Ca^2+^]_i_, indicating that the Ca^2+^ response through the activation of this channel is mediated solely by the influx of extracellular Ca^2+^. (**D**) The bar graph shows the statistical analysis of the Ca^2+^ response (expressed as a percentage) after TRPV4 activation in a medium without or with Ca^2+^. Bars represent the mean plus the standard error of the mean (S.E.M.), *n* = 5. Repeated-measures analysis of variance followed by Dunnett’s Multiple Comparison Test was performed. ** *p* < 0.01. The data in the graph bars are expressed as the percentage of the first stimulus with GSK101 (corresponding to 100% of the total Ca^2+^ response).

We first confirmed the presence of the TRPV4 channel in guinea pig tracheal smooth muscle by Western blot (Figure 1), as has been demonstrated in other species, such as human and mouse tracheal smooth muscle [46,47,51]. Our findings also demonstrated that TRPV4 is functionally expressed in guinea pig airway ASM cells, as shown in Figure 2. As we have previously reported, GSK101 caused a concentration-dependent increase in [Ca^2+^]_i_ with a slow onset and plateau phase, with the maximum Ca^2+^ increase occurring at 32 nM of this drug [49]. To confirm the specificity of this TRPV4 agonist, we used the antagonist GSK219. The addition of 100 nM GSK219, 5 min before stimulation of TRPV4 with 32 nM GSK101, significantly decreased (by ~94%) this agonist response (Figure 2A,B). Moreover, the perfusion of Ca^2+^-free Krebs solution completely blocked the increase in Ca^2+^ caused by pharmacological stimulation of TRPV4 (Figure 2C,D). These results suggest that the increase in [Ca^2+^]_i_ induced by TRPV4 agonist depends exclusively on external Ca^2+^ influx, with no involvement of internal Ca^2+^ stores.

### 2.2. Pharmacological TRPV4 Activation with GSK101 Mediates Extracellular Na^+^ Influx

**Figure 3:**
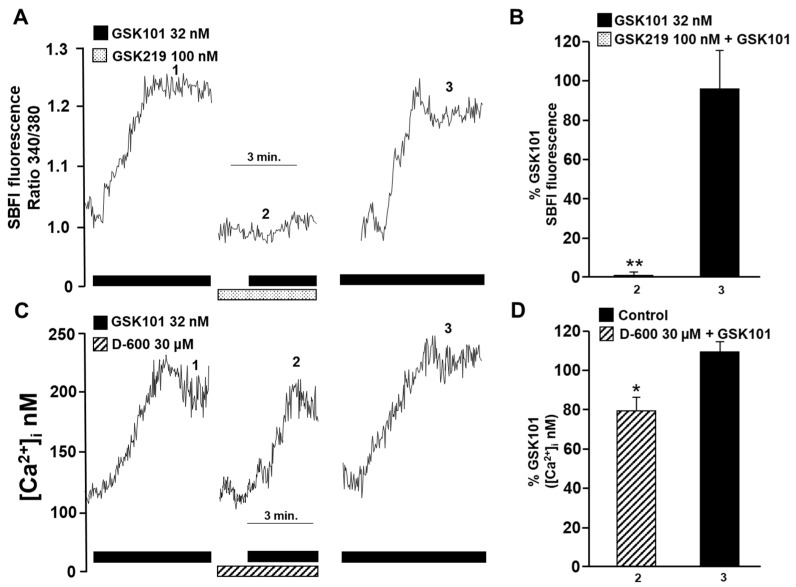
Possible involvement of TRPV4 in activating L-type voltage-dependent Ca^2+^ channels in guinea pig tracheal myocytes. (**A**) When 32 nM GSK101 (a selective TRPV4 agonist) was added, the fluorescence increased slowly and reached a plateau phase (1). This indicates that when TRPV4 is opened, Na^+^ influx into the cytoplasm is promoted. However, when cells were preincubated with GSK219 (a selective TRPV4 antagonist, 100 nM), the increase in fluorescence caused by a second stimulation with GSK101 (32 nM) was reduced by 99% (2). After the washout of the TRPV4 antagonist, a third stimulus with 32 nM GSK101 elicited a response similar to the first stimulus (3). (**B**) The graph shows the increase in SBFI-AM fluorescence (expressed as a percentage) caused by TRPV4 stimulation in the presence and after the use of the antagonist GSK219 (100 nM). (**C**) Methoxy verapamil (D-600, 30 µM), a blocker of L-type voltage-gated Ca^2+^ channels (L-VDCCs), significantly decreased the Ca^2+^ increase provoked by stimulation with the selective TRPV4 agonist (GSK101, 32 nM). The original record shows that the addition of 32 nM GSK101 caused an increase in [Ca^2+^]_i_, characterized by a slow onset and a plateau phase. After a second stimulus with the TRPV4 agonist, a 5 min preincubation with D-600 led to a significant decrease in the Ca^2+^ response by approximately 20%. These results suggest that TRPV4 opening causes Na^+^ influx into the cytoplasm of tracheal myocytes, which leads to depolarization of the plasma membrane and opening of L-VDCCs. (**D**) The bar graph depicts the inhibition expressed as a percentage of Ca^2+^ increase by adding D-600. * *p* < 0.05, ** *p* < 0.01 (compared with the control group of 4–5 experiments). Repeated-measures analysis of variance followed by Dunnett’s Multiple Comparison Test was performed. The data in the graph bars are expressed as the percentage of the first stimulus with GSK101 (corresponding to 100% of the total Ca^2+^ or Na^+^ response).

It is known that TRPV4 is a multimodal non-selective cation channel [59]. To determine whether this channel is permeable to other physiologically important cations in our model, such as Na^+^, we measured intracellular Na^+^ concentration ([Na^+^]_i_) using SBFI-AM, a sensitive Na^+^ fluorophore. The incubation of myocytes with 32 nM GSK101 resulted in a proportional increase in SBFI 340/380 fluorescence ratio, indicating an increase in [Na^+^]_i_ (Figure 3A). The addition of the TRPV4 antagonist (GSK219, 100 nM) abolished the increase in SBFI fluorescence caused by stimulation of the TRPV4 channel (Figure 3A,B).

Recently, we showed that the increase in [Ca^2+^]_i_ induced by TRPV4 activation was partially attenuated by the addition of D-600 (a blocker of L-VDCCs) in the Ca^2+^ plateau [49]. In the present work, pre-incubation with D-600 reduced the increase in [Ca^2+^]_i_ induced by TRPV4 activation by ~20% (Figure 3C,D). These findings indicate that the TRPV4 channel allows not only the influx of Ca^2+^ into ASM cells but also the influx of Na^+^. Additionally, the influx of Na^+^ through the TRPV4 channel may cause membrane depolarization and the opening of L-VDCCs. It is noteworthy that a significant portion of the Ca^2+^ response triggered by TRPV4 stimulation (approximately 20%) relies on the activation of L-VDCCs.

### 2.3. L-VDCCs Are Involved in Airway Smooth Muscle Contraction Mediated by TRPV4 Stimulation

**Figure 4:**
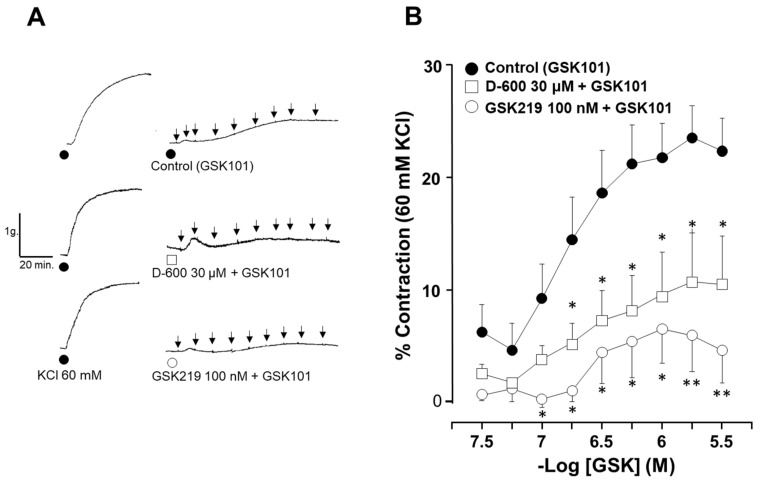
Contraction induced by TRPV4 activation is mediated in part by the opening of L-VDCCs. (**A**) The original traces show smooth muscle contraction induced by a cumulative concentration–response curve to TRPV4 agonist (GSK101) after a third stimulus with 60 mM KCl. The black arrows illustrate the addition of the different tested concentrations of GSK101 (32, 56, 100, 170, 320, 560, 1000, 1700, and 3200 nM). (**B**) The graph shows the cumulative effect of the administration of the TRPV4 agonist GSK101 (32–3200 nM). The antagonist GSK219 (100 nM) almost abolished the contraction response, while 30 µM D-600 significantly reduced the contraction response to GSK101. * *p* < 0.05, ** *p* < 0.01. *n* = 6–11. A one-way analysis of variance followed by Dunnett’s Multiple Comparison Test was performed.

We then examined whether activating TRPV4 can contract the airway smooth muscle. The cumulative addition of GSK101 at several concentrations induced a sustained contraction in guinea pig tracheal rings that reached approximately 24% of the 60 mM KCl contraction response (Figure 4). This contraction response was almost blocked with the TRPV4 antagonist GSK219 (Figure 3A,B). The possible role of L-VDCCs in the GSK101-induced ASM contraction in guinea pigs was investigated using an L-VDCC blocker. As can be seen in Figure 4B, the contractile response to GSK101 (170, 320, 560, 1000, 1700, and 3200 nM) was significantly reduced by 30 µM D-600. According to these findings, when TRPV4 is activated, it leads to the contraction of ASM by allowing the influx of Ca^2+^ and the opening of L-VDCCs caused by the influx of Na^+^. The opening of these channels results in the influx of Ca^2+^, which in turn contributes to the contraction of the ASM.

### 2.4. TRPV4 Blockade Decreases Airway Smooth Muscle Reactivity to Carbachol and Histamine

**Figure 5:**
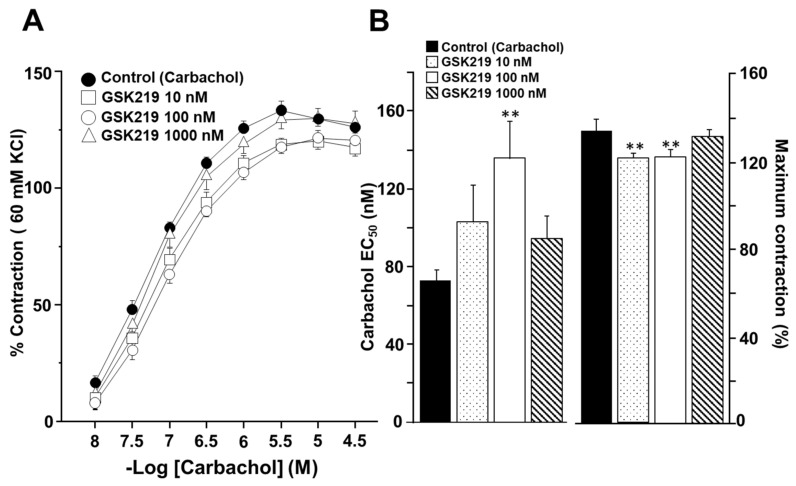
TRPV4 antagonist decreases carbachol (CCh) responsiveness in guinea pig tracheal smooth muscle. (**A**) Cumulative administration of CCh (10, 32, 100, and 320 nM, and 1 and 3.2 µM) caused concentration-dependent contraction of tracheal tissue (control group). Acute exposure of GSK219 at different concentrations (10, 100, and 1000 nM) shifted the concentration–response curve of CCh to the right and decreased the maximal contraction response. (**B**) Bar graphs show the analysis of effective concentration 50 (EC_50_) and maximum response of CCh. Symbols and bars illustrate mean ± S.E.M. Repeated measures analysis of variance followed by Dunnett’s multiple comparison test was performed. ** *p* < 0.01, *n* = 7.

Next, we investigated whether TRV4 activity is involved in the airway smooth muscle response to CCh and His, two well-known bronchoconstrictor agonists. As shown in Figure 5, CCh evoked a concentration-dependent contraction of guinea pig tracheal rings. Moreover, we found that the TRPV4 antagonist GSK219 at 10, 100, and 1000 nM shifted the cumulative concentration–response curve to CCh to the right (Figure 5A). After these data were analyzed, the CCh–EC_50_ when 100 nM GSK was used showed statistical differences compared with the control group carbachol (135.84 ± 19.66 nM vs. 72.75 ± 11.47 nM, respectively). The maximum CCh contraction was also significantly reduced by 10 and 100 nM GSK219 when compared to the control group (Figure 5B). Regarding the maximal contraction response, 10 nM GSK219 (120.90 ± 2.50%) and 100 nM GSK219 (121.87 ± 2.52%) showed similar values; i.e., the effect was not concentration dependent.

**Figure 6:**
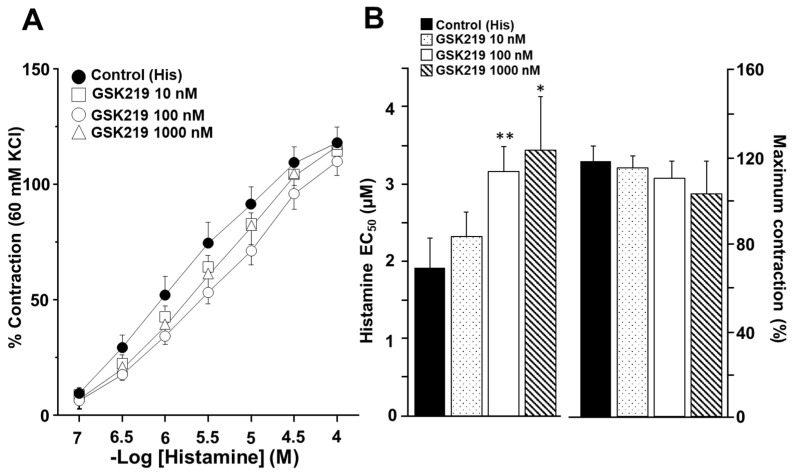
Pharmacological blockade of TRPV4 decreased the response to Histamine (His) during guinea pig tracheal contraction. (**A**) Cumulative administration of His (0.1, 0.32, 1, 3.2, 10, and 32 μM) elicited concentration-dependent contraction of guinea pig tracheal rings (control group). Different concentrations of GSK219 (10, 100, and 1000 nM) shifted the concentration–response curve relating to His to the right. (**B**) Bar graphs illustrate the analysis of the effective concentration 50 (EC_50_) and maximum response of His. Notice that the reduction in EC_50_ relating to His caused by GSK219 in the nanomolar range was concentration–dependent. Symbols and bars depict mean ± S.E.M. Repeated measures analysis of variance followed by Dunnett’s multiple comparison test was performed. * *p* < 0.05, ** *p* < 0.01, *n* = 7.

The cumulative concentration–response curve relating to His was also shifted to the right in the presence of the TRPV4 antagonist (Figure 6A). Significant differences in the EC_50_ of His were observed in the 100 (3.19 ± 0.30 µM) and 1000 nM (3.47 ± 0.72 µM) GSK219 groups compared to the control group (1.93 ± 0.39 µM). However, no significant differences were observed in maximum contraction caused by this agonist (Figure 6B). These results indicate that during CCh- or His-induced airway smooth muscle contraction, TRPV4 is activated, contributing to the contraction response.

### 2.5. TRPV4 Is Involved in Intracellular Ca^2+^ Increases Induced by Carbachol and Histamine

**Figure 7:**
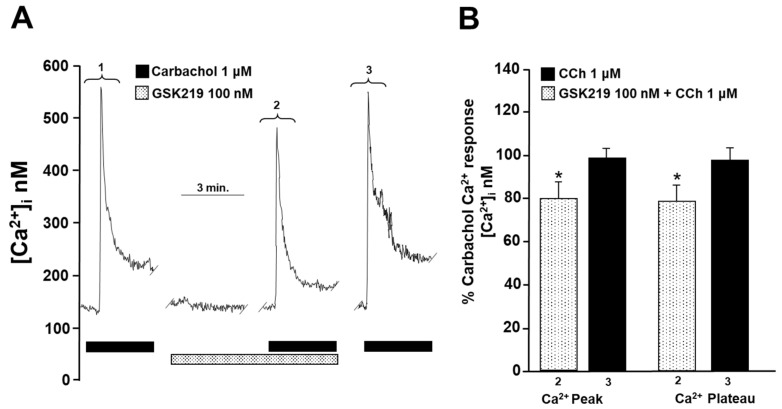
TRPV4 is activated during the stimulation of airway smooth muscle cells with carbachol. (**A**) Smooth muscle cells from the trachea were continuously perfused with Krebs solution at 37 °C. The addition of 1 µM carbachol (CCh) resulted in an increase in the intracellular Ca^2+^ concentration ([Ca^2+^]_i_), which was characterized by a rapid onset (a peak) at approximately 600 nM followed by a sustained plateau at around 75 nM (1). When a selective TRPV4 antagonist (100 nM GSK219) was administered for 5 min, it significantly reduced the CCh-induced increase in [Ca^2+^]_i_. The Ca^2+^ peak was reduced by approximately 22%, while the plateau was decreased by 24% in the presence of GSK219 in Krebs solution (2). After the TRPV4 antagonist was washed out, a third stimulation with 1 µM CCh produced a Ca^2+^ response similar to the first stimulation (3). (**B**) The graph illustrates the percentage of the Ca^2+^ response elicited by CCh stimulus in the presence and after use of the antagonist GSK219 (100 nM), *n* = 5. The use of GSK219 resulted in a significant reduction in the Ca^2+^ response elicited by CCh. * *p* < 0.05. Repeated measures analysis of variance followed by Dunnett’s multiple comparison test was performed. The data in the graph bars are expressed as the percentage of the first stimulus with CCh 1 µM (corresponding to 100% of the total Ca^2+^ response).

**Figure 8:**
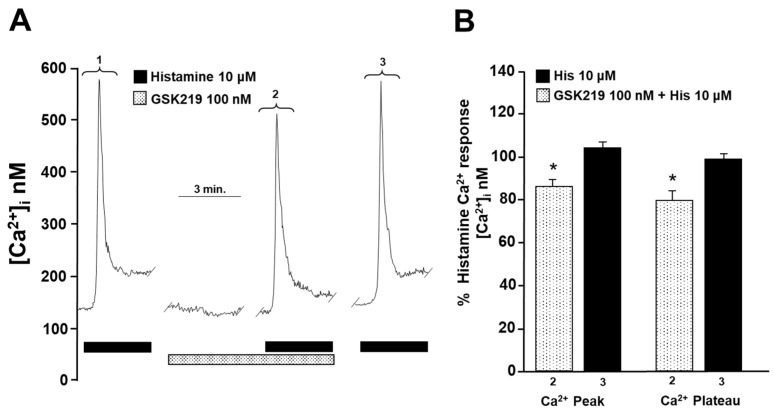
Blockade of TRPV4 decreases histamine-induced increase in intracellular Ca^2+^ concentration ([Ca^2+^]_i_) in guinea pig tracheal smooth muscle cells. (**A**) Single tracheal smooth muscle cells were constantly perfused with Krebs solution at 37 °C. The addition of 10 µM histamine (His) resulted in an increase in [Ca^2+^]_i_ characterized by a rapid onset (a peak) around 600 nM and a sustained plateau around 70 nM (1). Preincubation of cells with the selective TRPV4 antagonist (100 nM GSK219) decreased the peak Ca^2+^ and plateau by ~14% and ~21%, respectively, produced by a second stimulation with 10 µM His (2). After the washout of the TRPV4 antagonist, a third stimulation with 10 µM His produced a Ca^2+^ response similar to the first stimulation (3). (**B**) The graph illustrates the percentage of Ca^2+^ response elicited by His in the presence and after use of the antagonist GSK219 (100 nM), *n* = 5. * *p* < 0.05. Repeated measures analysis of variance followed by Dunnett’s multiple comparison test was performed. The data in the graph bars are expressed as the percentage of the first stimulus with 10 µM His (corresponding to 100% of the total Ca^2+^ response).

To investigate the cause of reduced contraction (induced by His or CCh) of guinea pig tracheal rings, we measured intracellular Ca^2+^ in isolated ASM cells. Single tracheal myocytes stimulated with CCh (1 µM) elicited a transient [Ca^2+^]_i_ increase (Ca^2+^ peak) that reached approximately 600 nanomoles, followed by a plateau of ~75 nanomoles. Adding 100 nM GSK219 5 min before CCh significantly reduced the Ca^2+^ peak and plateau triggered by this agonist by ~22% and ~24%, respectively. After washout of the TRPV4 antagonist, a subsequent CCh stimulation showed a similar Ca^2+^ response to that of the initial stimulation (Figure 7). Because our results showed that blockade of TRPV4 also decreased His-induced contraction, we examined the effect of GSK219 on His-evoked Ca^2+^ increase in ASM cells. In tracheal myocytes, 10 µM His induced a Ca^2+^ transient peak followed by a plateau that was reduced by TRPV4 antagonist (100 nM GSK219) by ~14 and ~21%, respectively. Washing away the antagonist restored the Ca^2+^ response induced by His (Figure 8). Based on our previous organ bath studies, we have chosen to use a concentration of 1 µM for CCh and 10 µM for His. These concentrations have been found to produce a contraction response of around 100% compared to the response elicited by KCl [30,60]. According to these findings, the contraction of tracheal tissues induced by GPCR stimulation is perceived by the TRPV4 channel, which is responsive to mechanical forces. The activation of this NSSC enables the entry of Ca^2+^, which is crucial for the optimal contraction of the ASM.

### 2.6. TRPV4 Blockade Also Lowers Smooth Muscle Reactivity to KCl

**Figure 9:**
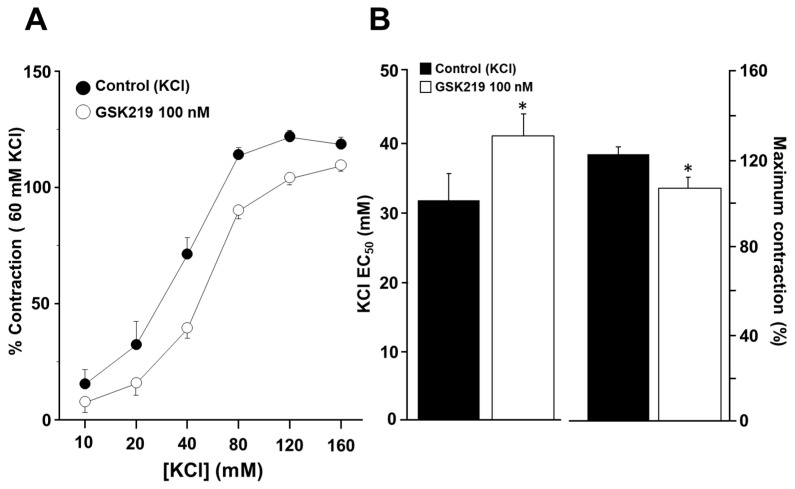
The TRPV4 antagonist GSK219 decreases the contraction response to KCl in guinea pig tracheal rings. (**A**) Cumulative administration of 10, 20, 40, 80, 120, and 160 mM KCl caused plasma membrane depolarization and subsequent contraction of tracheal tissues in a concentration-dependent manner (control group). Incubation of tracheal rings for 15 min with 100 nM GSK219 shifted the concentration–response curve of KCl to the right and decreased the maximal contraction response obtained by adding KCl. (**B**) Bar graphs depict the analysis of effective concentration 50 (EC_50_) and maximum response of KCl. Symbols and bars depict mean ± S.E.M. A Student’s *t*-test was performed for paired data (*n* = 6). * *p* < 0.05 compared with the control group.

We further explored whether TRPV4 could be activated by contraction of ASM induced by a chemical agent such as KCl. High concentrations of KCl have been commonly used to study smooth muscle contraction elicited by membrane depolarization. We observed that incubation of tracheal tissues with 100 nM GSK219 decreased smooth muscle contraction induced by increasing KCl concentrations (10, 20, 40, 80, 120, and 160 mM) (Figure 9). 

**Figure 10:**
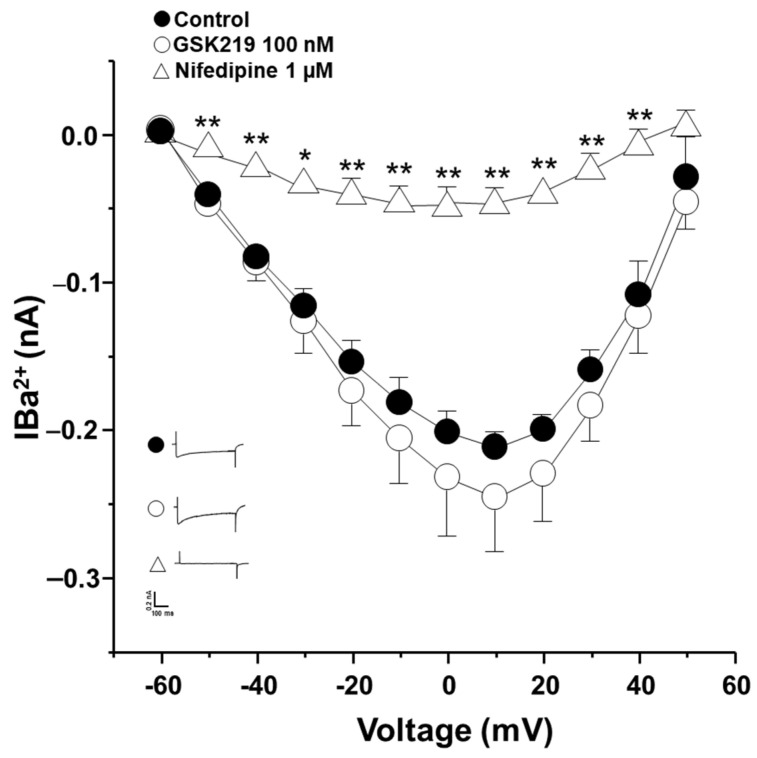
The TRPV4 antagonist (GSK219) does not alter Ca^2+^ currents in guinea pig airway smooth muscle cells. A protocol of depolarizing pulses from −60 to +50 mV in 10 mV steps produced Ba^2+^ inward currents that peaked at about 0.21 nanoamperes (nA). Ba^2+^ was used as an ion carrier instead of Ca^2+^ to enhance current responses through Ca^2+^ channels during the stimulus of depolarization. Application of 100 nM GSK219 did not alter Ba^2+^ currents. However, nifedipine, a blocker of L-type voltage-dependent Ca^2+^ channels, almost abolished them. Symbols and bars depict mean ± S.E.M. Repeated-measures analysis of variance followed by Dunnett’s test was performed. * *p* < 0.05, ** *p* < 0.01, *n* = 5.

Since KCl-induced contraction of guinea pig ASM is entirely dependent on membrane depolarization and hence external Ca^2+^ entry through L-VDCCs activation [40,61], we conducted patch-clamp experiments to rule out the possibility that GSK219 might block this channel. Electrophysiological recordings of Ca^2+^ currents showed that the TRPV4 antagonist at 100 nM did not alter L-VDCCs activity (Figure 10). To confirm the identity of this Ca^2+^ current, nifedipine (1 µM) was used; this blocker almost abolished Ca^2+^ currents (Figure 10). These findings indicate that mechanical forces occurring during ASM contraction activate TRPV4, independently of the bronchoconstrictor agonist tested. This activation results in the influx of extracellular Ca^2+^ through this channel and possibly through L-VDCCs, promoting an optimal contraction response.

### 2.7. Role of TRPV4 in Sarcoplasmic Reticulum Ca^2+^ Refilling

**Figure 11:**
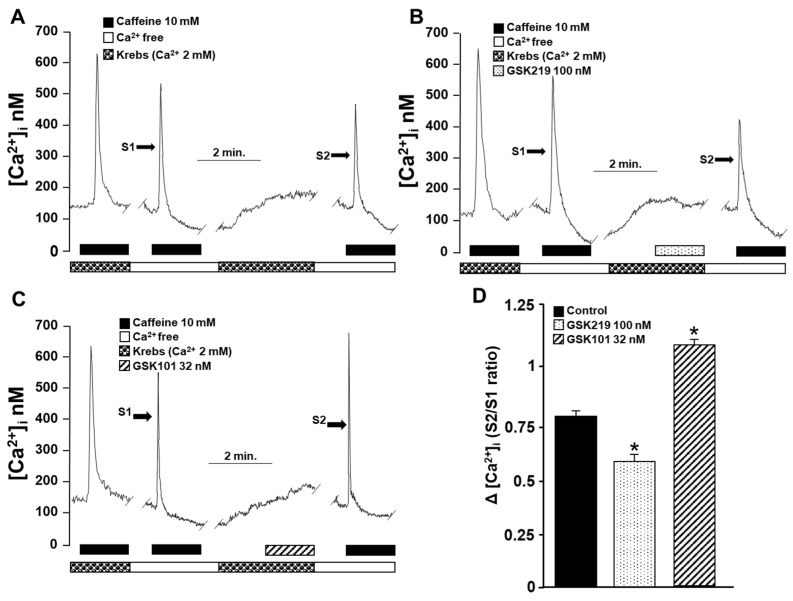
The role of TRPV4 in sarcoplasmic reticulum Ca^2+^ refilling was indirectly evaluated by an experimental method of repeated stimulation with caffeine in guinea pig tracheal myocytes. (**A**) Original fluorometric recording showing S1 as the first stimulation with caffeine (10 mM) in a Ca^2+^-free medium for 10 min (to promote depletion of SR) and S2 as the second response in a Ca^2+^-free medium. Replenishment of RS with Ca^2+^ was enabled by perfusing the cell with Krebs solution containing 2 mM Ca^2+^ after S1 and before S2 stimulation. (**B**) Blocking TRPV4 for 4 min with 100 nM GSK219 before stimulation with S2 decreased Ca^2+^ replenishment, while activation (**C**) with 32 nM GSK101 increased Ca^2+^ replenishment of SR. (**D**) Bar graph illustrating the decrease and increase in Ca^2+^ replenishment rate of SR, expressed as S2/S1 ratio, caused by TRPV4 blockade or activation, respectively. One-way analysis of variance, followed by Dunnett’s test, was performed. * *p* < 0.05, *n* = 4–5.

The above findings point out that TRPV4 increases [Ca^2+^]_i_ in airway myocytes mainly through the same channel and, to a lesser extent, through the opening of L-VDCCs. However, these Ca^2+^ increases do not result in a significant contractile response compared with that induced by CCh, His, or KCl. Therefore, we investigated the possibility that this increase in [Ca^2+^]_i_ might be involved in SR Ca^2+^ replenishment. The reuptake of Ca^2+^ by the SR is necessary for optimal contraction of the ASM [27,29,35,40,62,63]. This was investigated using an S2/S1 ratio described previously [35]. Under control conditions, myocytes showed an S2/S1 ratio of 0.81 ± 0.03 (Figure 11A,D), which decreased significantly when TRPV4 was blocked with 100 nM GSK219 (0.57 ± 0.06) (Figure 11B,D). In contrast, activating this channel with the agonist GSK101 (32 nM) increased the S2/S1 ratio (1.1 ± 0.04) (Figure 11C,D), demonstrating the role of TRPV4 in refilling the Ca^2+^ content of the SR.

## 3. Discussion

Our results show that guinea pig tracheal smooth muscle contraction triggered by classical bronchoconstrictor agonists such as carbachol and histamine or high K^+^ activates the polymodal TRPV4 channel. We observed that the blockade of TRPV4 during cumulative concentration–response curves relating to CCh, His, or KCl shifted the curve to the right and decreased the maximal contraction (only for CCh and KCl). We also found that, in guinea pig ASM cells, the activation of TRPV4 allowed Ca^2+^ and Na^+^ influx. Therefore, we hypothesize that the mechanical forces exerted by airway contraction stimulate TRPV4 opening, and Ca^2+^ influx facilitates the optimal response to the former agonists. In addition, Na^+^ influx through TRPV4 may depolarize the plasma membrane and trigger the opening of L-VDCCs, which could also contribute to the contractile response. The results also showed that this polymodal NSCC plays an important role in replenishing the Ca^2+^ of SR, which may enable Ca^2+^ availability for bronchoconstrictor agonists acting through the PLC-IP_3_ pathway.

ASM cells from species such as humans and mice express TRPV4 channels [46,47,49,51,53]. In this study, we confirmed the presence of this channel in the ASM of guinea pigs (Figure 1). TRPV4 allows the influx of divalent and monovalent cations such as Ca^2+^ and Na^+^, as we observed in Figure 2 and Figure 3. Na^+^ influx can lead to membrane depolarization, favoring an increase in [Ca^2+^]_i_ through the activation of other channels, including the L-VDCC. As previously reported, this channel would contribute to the sustained Ca^2+^ increases caused by the agonist GSK101 [34]. In this context, results in Figure 3 showed a decrease in TRPV4-induced Ca^2+^ increase when the L-VDCC blocker (D-600) was perfused before the GSK101 stimulus. Given these findings, activated TRPV4 could lead to smooth muscle contraction. We found that TRPV4 stimulation in tracheal rings resulted in a mild contractile response that was almost abolished by the antagonist GSK219 (Figure 4). Moreover, we found that this contraction was markedly diminished by the L-VDCC blocker D-600 (Figure 4). It has been demonstrated that activating TRPV4 in the ASM results in the production and release of ATP. This, in turn, triggers mast cells to release leukotrienes, leading to the contraction of the ASM [47,53]. However, our findings in Figure 4 suggest a new mechanism where TRPV4 stimulation leads to the influx of Ca^2+^ and Na^+^. The latter ion promotes the L-VDCCs opening, further increasing Ca^2+^ levels, ultimately causing ASM contraction.

Organ bath experiments were performed to investigate the role of TRPV4-induced Ca^2+^ increase in ASM contraction. In this regard, the activation of NSCCs may serve as a critical Ca^2+^ entry pathway required for ASM contraction induced by cholinergic agonists [34,64,65]. For example, we have shown that SOCC (a class of NSCCs) is one of the most significant membranal Ca^2+^ channels responsible for the sustained CCh-evoked contraction of the guinea pig ASM [34]. Similarly, several research groups have demonstrated that blockade of NSCCs relaxes mouse ASM precontracted with acetylcholine [41,42,43,61,66,67]. In this study, we used CCh and His to cause the contraction of tracheal tissues. We found that the TRPV4 antagonist 100 nM GSK219 significantly decreased the EC_50_ of CCh, while 100 and 1000 nM GSK219 reduced the EC_50_ of His (Figure 5B and Figure 6B). In addition, the antagonist GSK219 (10 and 100 nM) decreased the maximum contraction response induced by CCh but not by His (Figure 5 and Figure 6). These findings indicate the importance of the activation of TRPV4 during ASM contraction. NSCCs are essential to depolarize the plasma membrane and to open the L-VDCCs, as the role of voltage-dependent Na^+^ channels (VDNCs) in ASM is still controversial [68,69,70,71,72]. Thus, our results represent an additional mechanism exerted by TRPV4 to mediate a nonselective cation influx (Ca^2+^ and Na^+^) involved in the contraction of the ASM induced by CCh or His.

We then explored the underlying mechanisms of GSK219-mediated decreased CCh- and His-induced contraction. Intracellular Ca^2+^ measurements showed that the TRPV4 antagonist significantly reduced both the Ca^2+^ peak and the Ca^2+^ plateau induced by CCh or His (Figure 7 and Figure 8). The Ca^2+^ peak is generated by the release of Ca^2+^ from the SR via the IP_3_ receptor, and the plateau is mediated by Ca^2+^ entry via the NSCCs and the L-VDCCs [34]. In this sense, a physical and functional coupling between TRPV4 and the IP_3_ receptor in mouse ASM cells is suggested [52]. CCh-induced activation of the IP_3_ receptor leads to the activation of TRPV4 and subsequent Ca^2+^ influx [52]. This phenomenon may also explain why the Ca^2+^ and contraction response triggered by CCh is diminished when TRPV4 is blocked. Furthermore, it is known that His-induced contraction of the ASM is mediated by many pathways, such as the IP_3_ signaling pathway and activation of the L-VDCC after the stimulation of the H_1_ receptor. As studied in bovine, canine, and guinea pig ASM, the histamine response depends primarily on L-VDCCs and not on the IP_3_ signaling cascade [29,62,63], contrariwise to CCh, where L-VDCCs play a minor role [27,34,35]. The absence of effect of GSK219 on the maximal response elicited by histamine and the minor effect of this blocker on the His-Ca^2+^ response compared to CCh (Figure 7 and Figure 8) might suggest a physical coupling between the TRPV4 channel and IP_3_ receptor. However, further studies are needed to confirm this hypothesis.

Given our experimental results and the possible interaction of TRPV4 with the IP_3_ receptor, we wondered whether TRPV4 might also be implicated in the ASM contractions that do not involve the PLC-IP_3_ pathway. Our experiments on tracheal rings showed that blockade of TRPV4 also decreased the EC_50_ and the maximal response of high K^+^-induced contraction (Figure 9). In this context, it is known that KCl contraction is entirely dependent on the opening of L-VDCCs [40]; i.e., KCl depolarizes the smooth muscle membrane that activates L-VDCCs, leading to the entry of Ca^2+^, eventually causing contraction. We conducted patch-clamp experiments to rule out the possibility that GSK219 could block the L-VDCC and thus reduce the contraction induced by KCl. We found that GSK219 did not alter nifedipine-sensitive Ca^2+^ currents in cultured tracheal myocytes (Figure 10), ruling out a nonspecific effect of this drug.

**Figure 12:**
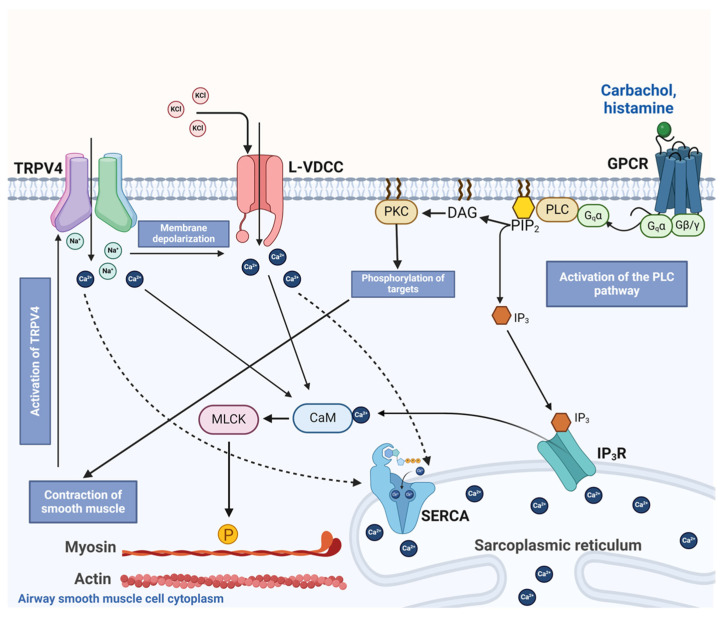
Schematic representation of the proposed role of TRPV4 in airway smooth muscle contraction. Bronchoconstrictor agonists such as carbachol or histamine occupy their respective G protein-coupled receptor (GPCR), inducing the disassembly of the trimeric Gq protein into subunits α and β/γ. Later, Gqα activates phospholipase C (PLC) to induce the formation of inositol triphosphate (IP_3_) and diacylglycerol (DAG). IP_3_ stimulates its receptor (IP_3_R) in the sarcoplasmic reticulum (SR), driving the release of Ca^2+^ into the cytoplasm, while DAG activates protein kinase C (PKC), which phosphorylates targets involved in smooth muscle contraction. In the cytoplasm, Ca^2+^ binds to calmodulin (CAM), triggering myosin light chain kinase (MLCK) action. This kinase phosphorylates the regulatory chain of myosin, inducing the cross-bridge cycle with actin and smooth muscle contraction in the airway. Chemical agents such as KCl also produce smooth muscle contraction by causing membrane depolarization and opening the L-type voltage-dependent Ca^2+^ channel (L-VDCC). This channel allows the influx of Ca^2+^ into the cytoplasm and the subsequent contraction. Contractile stimulus is detected by TRPV4, which in turn triggers the influx of Ca^2+^ and Na^+^. This latter ion favors membrane depolarization and the opening of the L-VDCC. Ca^2+^ entering through TRPV4 and L-VDCC are taken up into the SR via the sarcoplasmic Ca^2+^ ATPase (SERCA), contributing to the refilling of Ca^2+^ of this organelle. The activation of TRPV4 and Ca^2+^ and Na^+^ influx through this polymodal channel participates in the optimal contraction of airway smooth muscle.

NSCCs are important for replenishing internal Ca^2+^ stores. Reuptake of Ca^2+^ by the SR optimizes ASM contraction [27,29,35,40,62,63]. To evaluate whether TRPV4 was involved in the refilling of Ca^2+^ of the SR, we used an S2/S1 protocol in which we pharmacologically blocked or activated this polymodal channel. Our results showed that the antagonist of TRPV4 decreased the SR replenishment. In contrast, the activation of TRPV4 significantly increased the SR Ca^2+^ refilling (Figure 11). This effect on SR replenishment could also be due to the opening of L-VDCCs caused by Na^+^ influx via TRPV4 channels. In this context, it is known that L-VDCCs are important channels involved in the replenishment of the Ca^2+^ content of the SR [35]. Moreover, agonist-induced, sustained, and optimal contraction requires a continuous Ca^2+^ influx into the SR to maintain adequate levels of this ion. The blockade of TRPV4 during CCh- or His-induced tracheal contraction could lead, in part, to reduced Ca^2+^ content in the SR. This could partly explain the reduced contraction response observed in Figure 5 and Figure 6. Collectively, our results indicate that mechanical forces occurring during ASM contraction activate TRPV4, resulting in extracellular Ca^2+^ influx through this channel and possibly through L-VDCCs. This promotes the refilling of Ca^2+^ of the SR and the optimal contraction response (Figure 12). Once TRPV4 is activated during contraction, the extent to which Ca^2+^ enters through this channel and via the L-VDCCs and its contribution to the contraction induced by His or CCh needs to be explored. However, it appears that only 15–20 percent of the Ca^2+^ increase produced by TRPV4 activation corresponds to the L-VDCCs activity (Figure 3C,D) [49].

In the context of lung diseases, TRPV4 is involved in the response of ASM cells to mechanical stress, which has been linked to dysfunction, i.e., hyperreactivity of this tissue [48,49,50,58,73,74,75]. For instance, some people with asthma are more sensitive to hypotonic stimuli than others. Researchers have found that a specific single nucleotide polymorphism in the TRPV4 gene, known as rs6606743, is markedly linked to the development of airway osmotic hyperresponsiveness in asthma patients with uncontrolled bronchial asthma, as compared to control patients who did not show any response to bronchoprovocation [54]. Also, it has been found that the polymorphisms of the TRPV4 gene were significantly associated with COPD features, as determined through single nucleotide polymorphism analyses [55]. Diesel exhaust particles induced a long-lasting influx of Ca^2+^ ions in human respiratory epithelial cells via TRPV4. This effect is enhanced by the human genetic polymorphism TRPV4_P19S_, which predisposes to COPD [76].

In cell types other than ASM cells, such as macrophages and lung epithelial cells, mechanical stretching can trigger the release of pro-inflammatory cytokines by activating an influx of Ca^2+^ that relies on the activity of the TRPV4 [77]. Moreover, inhibiting TRPV4 reduced the release of pro-inflammatory cytokines and increased lung barrier permeability in a mouse model of high tidal volume breathing [77]. TRPV4 detects damage to epithelial junctions in the airways and rapidly triggers a Ca^2+^-dependent cellular response that augments inflammation [47,78]. An increased expression of TRPV4 in bronchial epithelial cells has been linked to a higher risk of sensitization to fungal allergens and asthma [78]. Administration of the TRPV4 antagonist HC-067047 reduced levels of interleukin (IL)-4, IL-3, and STAT6 in guinea pigs sensitized to ovalbumin (OVA) [17]. In a mouse model of allergic asthma induced by OVA, the TRPV4-p38 MAPK pathway is activated [79]. Exposure to formaldehyde (a chemical broadly used in household products) and high relative humidity simultaneously intensified the activation of the TRPV4-p38 MAPK pathway, leading to worsening airway inflammation and increased mucus secretion [79]. It has also been shown that blocking TRPV4 activity and Ca^2+^ influx in lung fibroblasts can effectively block the fibrotic process triggered by mechanical or inflammatory signals and alleviate pulmonary fibrosis in COPD [80]. Furthermore, in human bronchial epithelial cells, the TRPV4 channel can sense changes in volume. This can activate Ca^2+^-activated K^+^ channels, regulating cell volume in response to osmotic stress [81].

In addition, it has been suggested that the TRPV4 channel is involved in the proliferation of bronchial smooth muscle cells, which is linked to chronic asthma. When Ca^2+^ enters the cell through this channel, it activates the intermediate conductance Ca^2+^-activated potassium channel, K_Ca_3.1 [46]. This channel is known to be a significant regulator of cell proliferation and differentiation. Considering the multiple roles of TRPV4 in the respiratory tract function, targeting TRPV4 could be an effective approach to treat respiratory diseases like asthma or COPD, which involve mechanical stress on several entities such as ASM cells, epithelial, and inflammatory cells in the airways. However, there is a need for further pharmacologic and clinical studies to fully comprehend the molecular mechanism of TRPV4 in lung disease development.

## 4. Materials and Methods

### 4.1. Experimental Animals

We used male Hartley guinea pigs weighing 300–450 g bred under standard conditions in our institutional animal facilities (filtered air at 21 °C, 50–70% humidity, and sterilized bed) and fed Harlan^®^ pellets and sterilized water. The guinea pig is one of the most frequently used experimental models for asthma studies [82]. Many of the key physiological processes and chemical mediators in the pathogenesis of this disease were first demonstrated in the guinea pig, such as the action of histamine and leukotrienes [82,83,84,85]. The histamine H_1_ receptor and the cysteinyl leukotriene (cysLT)-1 receptor are partially responsible for the acute, allergic hypersensitivity reactions in asthmatics’ airways. These reactions include ASM contraction, eosinophil infiltrate, airway hyperresponsiveness, and mucus production [84,86,87]. The same receptor activations are observed in guinea pigs [87,88]. In addition, guinea pig airway smooth muscle pharmacology is more similar to human ASM pharmacology than most other commonly used species. Bronchoconstrictor and bronchodilator agonists have almost identical potency and efficacy in humans as in guinea pigs [82,89]. The Scientific and Bioethical Committees of the Faculty of Medicine at the National Autonomous University of Mexico approved the protocol. The American Physiological Society’s https://www.physiology.org/career/policy-advocacy/policy-statements/care-and-use-of-vertebrate-animals-in-research?SSO=Y, 2014, (accessed on 11 November 2023) and the National Institutes of Health’s published guidelines for the care and use of laboratory animals (NIH, Eight Edition, National Academies Press, 2011, ISBN-13:978-0-309-15400-0), as well as the Mexican National Animal Welfare Laws and Protection and the General Health Law for Health Research (NOM-062-Z00-1999), were all followed when conducting the experiments.

### 4.2. Intracellular Ca^2+^ Measurements in Tracheal Myocytes

Pentobarbital sodium (50 mg/kg) was administered intraperitoneally to anesthetize the animals before exsanguination. Tracheas were removed and placed in a plate containing modified Krebs–Henseleit solution with the following composition (in mM): 118 NaCl, 25 NaHCO_3_, 4.6 KCl, 1.2 KH_2_PO_4_, 1.2 MgSO_4_, 2 CaCl_2_, 11 glucose [30,34,90,91,92,93,94]. Tissues were dissected and freed from connective tissue and epithelium. To isolate guinea pig tracheal myocytes, smooth muscle strips were placed in 5 mL Hanks’ solution (GIBCO, Waltham, MA, USA) containing 2 mg L-cysteine and 0.04 U/mL papain. 1 M NaHCO_3_ was used to adjust the pH to 7.4, and the preparations were then incubated at 37 °C for 10 min. The excess enzyme was then removed with Leibovitz solution (L-15, GIBCO), and the tissues were placed in Hanks’ solution containing 1 mg/mL collagenase type I and 1 mg/mL collagenase type II for 10 min at 37 °C. The smooth muscle strips were gently dispersed by mechanical shaking until detached cells were seen. L-15 was used again to stop enzymatic activity, and cells were centrifuged at 800 rpm for 5 min at 20 °C, with the supernatant discarded. This last step was repeated once.

Tracheal myocytes were loaded with 2.5 µM fura 2-AM and maintained in low Ca^2+^ (0.1 mM) at room temperature (~22 °C) for 1 h. The loaded cells were placed in a heated perfusion chamber with a glass cover at the bottom. The heated perfusion chamber was mounted on an inverted microscope (Diaphot 200, Nikon, Tokyo, Japan). Myocytes attached to the glass cover were perfused at approximately 2 mL/min with Krebs solution at 37 °C, with 5% CO_2_ in 95% oxygen and a pH of 7.4. Fura 2-AM-loaded myocytes were alternately irradiated with pulses of 340 and 380 nm excitation light, and emission light was detected at 510 nm using a Photon Technology International model D-104 microphotometer (PTI, Princeton, NJ, USA). [Ca^2+^]_i_ was calculated using the Grynkiewicz formula [95], and fluorescence was measured at 0.5 s intervals. The Kd of fura 2-AM was estimated to be 386 nM [96]. To calibrate the system, cells were exposed to 10 µM ionomycin in Krebs solution containing 10 mM Ca^2+^ and to 10 mM EGTA in Ca^2+^-free Krebs solution to obtain the mean 340 to 380 nm fluorescence ratios for R_max_ and R_min_, respectively. Under these conditions, R_min_ was 0.39 and R_max_ was 6.06. At 380 nm light excitation, the fluorescence ratio in both Ca^2+^ saturated cells and Ca^2+^ free media was 4.23. The recordings were stored on a microcomputer, and the data acquisition and analysis software (Felix version 1.21; PTI) was used for analysis.

To evaluate and confirm the presence of the TRPV4 channel in our model, resting-state single tracheal myocytes were stimulated with GSK101, a selective TRPV4 agonist [97], at a concentration of 32 nM. Some cells were perfused with the TRPV4 antagonist GSK219 (100 nM) [47] or with Krebs solution without Ca^2+^ and with 0.1 mM EGTA before the GSK101 pulse (32 nM). This was performed to assess the specificity of the TRPV4 agonist and to determine whether the Ca^2+^ response of this agonist was solely dependent on the influx of external Ca^2+^. In another series of experiments, cells were treated with the blocker of L-VDCCs, D-600 (30 µM), before stimulation with GSK101, to study a possible role of TRPV4 channel in the membrane depolarization and activation of L-VDCCs. In additional experiments, tracheal smooth muscle cells were perfused with 100 nM GSK219 prior to stimulation with histamine 10 µM or carbachol 1 µM to investigate the role of TRPV4 in the Ca^2+^ response elicited by bronchoconstrictors that activate the PLC-IP_3_ pathway.

We further examined the ability of myocytes to replenish their internal Ca^2+^ stores to assess the role of TRPV4-mediated Ca^2+^ entry on the SR-Ca^2+^ content. As previously described, we performed an S1/S2 ratio protocol [29,35,40]. Briefly, the viability of single cells was evaluated by stimulation with 10 mM caffeine in Krebs solution before the experimental protocol. Myocytes were then perfused with a Ca^2+^-free solution, and 10 mM caffeine (S1) was added for 10 min. We have previously shown that SR Ca^2+^ stores are entirely depleted after 10 min of caffeine stimulation in a Ca^2+^-free medium [63]. After washing with a Ca^2+^-free medium to remove caffeine, Krebs solution containing 2 mM Ca^2+^ was added to the cells for 10 min to allow SR Ca^2+^ to replenish. We have demonstrated that this period can sufficiently replenish SR to about 70% [29,40]. After all, caffeine stimulation (S2) was repeated under the same Ca^2+^-free Krebs solution. The degree of SR Ca^2+^ replenishment was determined by the S2/S1 ratio in these tests [29,35,40]. In some of the experiments reported here, cells were stimulated 4 min before S2 with GSK101 (32 nM, for 3 min) or 100 nM GSK219 (for 3 min).

### 4.3. Organ Baths

The same procedure described above was used to obtain tracheal tissues from guinea pigs. Tracheas freed from connective tissue were cut into eight rings and suspended in a 5 mL organ bath filled with Krebs solution at 37 °C. To maintain the pH at 7.4, the preparations were constantly bubbled with 5% CO_2_ in oxygen. Each tracheal ring was attached with a silk thread to an isometric force transducer (model FT03; Grass Instruments, West Warwick, RI, USA) connected to an analog-to-digital interface (Digidata 1440A; Axon) and a signal conditioner (CyberAmp 380; Axon Instruments, Foster City, CA, USA). AxoScope, version 10.2 from Axon, was used to record and analyze the data. At the beginning of these tests, tissues were subjected to a resting tension of 1 g for 30 min. Three consecutive stimulations with KCl (60 mM) were applied to condition the tissues and optimize the smooth muscle contractile apparatus. To investigate whether the activation of TRPV4 could trigger ASM contraction by increasing Ca^2+^ influx, a cumulative concentration–response curve was generated for GSK101 (32, 56, 100, 170, 320, 560, 1000, 1700, and 3200 nM) under three conditions: in the absence of any drug (control), in the presence of GSK219 (100 nM), and in the presence of D-600 (30 µM). D-600 was used to examine the potential involvement of L-VDCCs in ASM contraction caused by TRPV4 stimulation. In another set of experiments, cumulative concentration–response curves relating to histamine (His, 100 nM to 100 mM), carbachol (CCh, 10 nM to 32 mM), or KCl (10, 20, 40, 80, 120, and 160 mM) were generated in the absence (control) or continuous presence of 10, 100, or 1000 nM GSK219 incubated for 15 min. This procedure allowed us to examine the participation of TRPV4 during the ASM contraction (a mechanical stimulus) caused by classical bronchoconstrictor agonists and a chemical substance such as KCl. The eight rings that could be isolated from a single trachea were each subjected to a separate experimental design.

### 4.4. Patch-Clamp Recordings

Guinea pig tracheal myocytes were isolated using the procedure described above and then cultured after resuspending the cell pellet in minimal essential medium (MEM, GIBCO) containing 5% fetal bovine serum, 2 mM L-glutamine, 10 U/mL penicillin, 10 mg/mL streptomycin, and 10 mM glucose. Cells were cultured for 48 h at 37 °C in an incubator containing 5% CO_2_ after being seeded onto round coverslips coated with sterile rat tail collagen.

In a 1 mL perfusion chamber, myocytes on the coverslips were submerged and settled to the bottom. This chamber was perfused with an external solution containing Ba^2+^ to replace Ca^2+^ as an inward charge carrier at a gravity flow rate of 1.5–2.0 mL/min to record Ca^2+^ currents. The external solution contained in mM: 136 NaCl, 6 CsCl, 5 BaCl_2_, 11 glucose, 10 HEPES, and 0.1 niflumic acid, reaching a pH of 7.4 with CsOH. Patch-clamp studies were performed at room temperature (~22 °C). An Axopatch 200A amplifier (Axon Instruments) was used to record Ca^2+^ currents triggered by depolarizing voltage steps (voltage clamp). A horizontal micropipette puller (P-87, Sutter Instruments Co, Novato, CA, USA) was used to fabricate patch pipettes from 1B200F-6 glass (WordPrecision Instruments, Sarasota, FL, USA). The resistance of these pipettes before sealing the smooth muscle cells ranged from 2 to 5 ΩM. The composition of the internal solution was (mM): 130 CsCl, 2 MgCl_2_, 10 HEPES, 10 EGTA, 3.6 ATP disodium salt, and 1.9 GTP sodium salt, with a pH of 7.3 adjusted with CsOH. The currents were filtered at 1–5 KHz and digitized at 10 KHz using a Digidata 1440A (Axon Instruments). The original recordings were stored on a computer and then analyzed using pClamp v10.2 software (Axon Instruments).

To examine Ca^2+^ currents, a series of conditioned depolarizing pulses with potentials ranging from −60 to +50 mV were applied to tracheal myocytes in 10 mV steps during 500 ms and 1 Hz, starting from a holding potential of −60 mV. Following the control protocol, the same experimental protocol was performed with the addition of 100 nM GSK219 or 1 µM nifedipine (to characterize the nature of the L-type Ca^2+^ current). The maximum current peak at each measured voltage was used to analyze the current changes.

### 4.5. Intracellular Na^+^ Measurements in Tracheal Myocytes

We measured intracellular Na^+^ concentration [Na^+^]_i_ as previously described by our research group. After collection, tracheal myocytes were incubated for 3 h at room temperature (22–25 °C) with 15 µM SBFI-AM (Thermofisher, Waltham, MA, USA) and 0.075% pluronic acid (Sigma, St. Louis, MO, USA). Then, SBFI-AM-loaded cells were placed in a perfusion chamber for 30 min to allow them to attach to the bottom of the chamber. During this time, Krebs solution was continuously perfused into the cells. Like the intracellular Ca^2+^ measurement experiments, cells loaded with SBFI-AM were irradiated with alternating pulses of 340 and 380 nm excitation light. Emission light was collected at 510 nm using a PTI model D-104 microphotometer.

Single airway smooth muscle cells were stimulated with TRPV4 agonist (GSK101, 32 nM) to observe Na^+^ entry through this channel. In some experiments, the antagonist of this receptor, GSK219 (100 nM), was perfused 5 min before TRPV4 stimulation with GSK101 to determine the specificity of this agonist.

### 4.6. Western Blotting for TRPV4

Guinea pig tracheal smooth muscle strips were collected, frozen in liquid nitrogen, and stored until assayed. A Polytron PT3100 (Kinematica, Lucerne, Switzerland) was used to homogenize each tissue in 50 μL RIPA lysis buffer (Santa Cruz Biotechnology, cat. no. sc-24948, Santa Cruz, CA, USA) containing protease inhibitor cocktail (Sigma, cat. no. P8340). The homogenized samples were centrifuged at 3000 rpm and 4 °C for 15 min. A commercial kit (RC DC Protein Assay, catalog 500–0119, Bio-Rad, Hercules, CA, USA) was used to measure total protein concentration. Samples were split into different lanes of 10% SDS-polyacrylamide gel and electrophoresed under reducing conditions. Proteins were transferred to a Bio-Rad polyvinylidene fluoride membrane and blocked with 5% nonfat dry milk in PBS tween (Tween 20, 0.1%) for two hours at room temperature. A rabbit polyclonal antibody (#ACC-034; 1:200) prepared against TRPV4 was incubated on the membranes at 4 °C for an entire night. Subsequently, the membranes were treated for two hours at room temperature with a goat anti-rabbit IgG secondary antibody (1:5000) coupled with horseradish peroxidase. An improved chemiluminescent reactant (Luminol; Santa Cruz Biotechnology, cat. no. sc-2048 CA, USA) was used to generate immunoblots. Kodak Digital Science ID software, version 2.03 (Eastman Kodak, New Haven, CT, USA), was used to perform densitometric analysis on TRPV4 immunoblots.

### 4.7. Drugs and Chemicals

**Figure 13:**
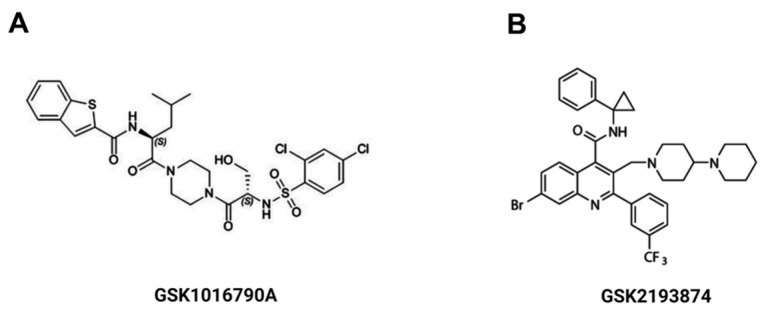
Chemical structure of the TRPV4 agonist, GSK1016790A (**A**) and the TRPV4 antagonist GSK2193874 (**B**).

GSK1016790A (GSK101, TRPV4 agonist), GSK2193874 (GSK219, TRPV4 antagonist), Methoxyverapamil (D-600), nifedipine, histamine and carbamylcholine chloride (carbachol) were all purchased from Sigma Chem. Co. (St. Louis, MO, USA). Absolute ethanol was used to dilute D-600 and nifedipine; the highest percentage used was 0.1% *v*/*v* of the vehicle. GSK101 and GSK219 were dissolved in DMSO 0.1% *v*/*v*. Chemical structures of GSK101 and GSK219 are illustrated in Figure 13.

### 4.8. Statistical Analysis

Repeated-measures analysis of variance followed by Dunnett’s multiple comparisons test were used to analyze the results of the [Ca^2+^]_i_ and [Na^+^]_i_ experiments. Patch-clamp results were also analyzed using these statistical tests. The organ bath data were analyzed using a one-way analysis of variance followed by Dunnett’s comparison tests or paired Student’s *t*-test. Each “*n*” value in the organ bath and isolated myocyte studies represents a single animal. Data are presented as mean ± the standard error of the mean (S.E.M.) in this manuscript’s figures. The threshold for statistical significance was set bimarginally at *p* < 0.05.

## 5. Conclusions

Our studies show that TRPV4 is activated during guinea pig tracheal smooth muscle contraction triggered by CCh, His, or KCl. The activation of this channel mediates Na^+^ and Ca^2+^ influx, which contributes to SR refilling and optimal tissue contraction. Our research provided evidence that TRPV4 may play a role in plasma membrane depolarization and opening of L-VDCCs.

## Data Availability

The data are available upon request at the electronic address jreyes@facmed.unam.mx.
